# 加速溶剂萃取-固相萃取净化结合超高效液相色谱-串联质谱法测定沉积物中双酚类化合物

**DOI:** 10.3724/SP.J.1123.2022.12015

**Published:** 2023-07-08

**Authors:** Qiuxu WANG, Qiyan FENG, Xueqiang ZHU

**Affiliations:** 中国矿业大学环境与测绘学院,江苏徐州221116; School of Environment Science and Spatial Informatics, China University of Mining and Technology, Xuzhou 221116, China

**Keywords:** 加速溶剂萃取, 固相萃取, 超高效液相色谱-串联质谱, 双酚类化合物, 沉积物, 正交试验, accelerated solvent extraction (ASE), solid-phase extraction (SPE), ultra performance liquid chromatography-tandem mass spectrometry (UPLC-MS/MS), bisphenols, sediment, orthogonal test

## Abstract

双酚类化合物(bisphenols)属于内分泌干扰物,具有生物累积性、持久性和雌激素活性,较低含量的双酚类化合物即会对人体健康和生态环境产生不利影响。为了准确检测沉积物中的双酚类化合物,本工作建立了加速溶剂萃取-固相萃取净化结合超高效液相色谱-串联质谱检测沉积物中双酚A(BPA)、双酚B(BPB)、双酚F(BPF)、双酚S(BPS)、双酚Z(BPZ)、双酚AF(BPAF)、双酚AP(BPAP)7种双酚类化合物的方法。优化了7种双酚类化合物的质谱参数,比较了在3组不同流动相条件下,7种双酚类化合物的响应值、分离效果和色谱峰形状。沉积物样品使用加速溶剂萃取方法进行前处理,采用正交试验优化了萃取溶剂、萃取温度和循环次数。实验结果表明,采用Acquity UPLC BEH C_18_色谱柱(100 mm×2.1 mm, 1.7 μm),以0.05%(v/v)氨水和乙腈为流动相进行梯度洗脱,可以实现7种双酚类化合物的快速分离;经正交试验优化后的萃取条件如下:萃取溶剂为乙腈,萃取温度为100 ℃,循环次数为3次。7种双酚类化合物在1.0~200 μg/L内线性关系良好(相关系数(*r*^2^)均大于0.999),检出限为0.01~0.3 ng/g。在3个加标水平(2.0、10、20 ng/g)下,7种双酚类化合物的回收率为74.9%~102.8%,相对标准偏差为6.2%~10.3%(*n*=3)。应用该方法分析了骆马湖湖区及其入湖河流沉积物中7种双酚类化合物的含量,结果表明:在骆马湖湖区沉积物中检测出BPA、BPB、BPF、BPS、BPAF,入湖河流沉积物中检测出BPA、BPF、BPS;其中BPA、BPF的检出率为100%,沉积物中BPA、BPF的含量分别为11.9~38.0 ng/g和11.0~27.3 ng/g。该方法简便、快速,准确度和精密度较高,适用于沉积物中7种双酚类化合物的检测。

## Abstract (en)

Bisphenols are endocrine disruptors that are characterized with bioaccumulation, persistence, and estrogenic activity. Even low contents of bisphenols can exert adverse effects on human health and the ecological environment. Herein, a method combining accelerated solvent extraction and solid-phase extraction purification with ultra performance liquid chromatography-tandem mass spectrometry was developed for the accurate detection of bisphenol A (BPA), bisphenol B (BPB), bisphenol F (BPF), bisphenol S (BPS), bisphenol Z (BPZ), bisphenol AF (BPAF), and bisphenol AP (BPAP) in sediments. The mass spectrometric parameters of the seven bisphenols were optimized, and the response values, separation effects, and chromatographic peak shapes of the target compounds were compared under three different mobile phase conditions. The sediment samples were pretreated by accelerated solvent extraction, and orthogonal tests were used to optimize the extraction solvent, extraction temperature, and cycle number. The results showed that the use of 0.05% (v/v) ammonia and acetonitrile as the mobile phase for gradient elution could rapidly separate the seven bisphenols on an Acquity UPLC BEH C_18_ column (100 mm×2.1 mm, 1.7 μm). The gradient program was as follows: 0-2 min, 60%A; 2-6 min, 60%A-40%A; 6-6.5 min, 40%A; 6.5-7 min, 40%A-60%A; 7-8 min, 60%A. Orthogonal experiments indicated that the optimal extraction conditions were as follows: extraction solvent of acetonitrile, extraction temperature of 100 ℃, and cycle number of three. The seven bisphenols showed good linearity in the range of 1.0-200 μg/L, with correlation coefficients (*r*^2^) greater than 0.999, and the limits of detection were 0.01-0.3 ng/g. The recoveries for the seven bisphenols ranged from 74.9% to 102.8% at three spiking levels (2.0, 10, 20 ng/g), with relative standard deviations ranging from 6.2% to 10.3%. The established method was applied to detect the seven bisphenols in sediment samples collected from Luoma Lake and its inflow rivers. BPA, BPB, BPF, BPS, and BPAF were detected in the sediments of the lake, and BPA, BPF, and BPS were detected in the sediments of its inflow rivers. The detection frequency of BPA and BPF was 100%, and the contents of these bisphenols in the sediment were 11.9-38.0 ng/g and 11.0-27.3 ng/g, respectively. The developed method is simple, rapid with high accuracy and precision, and is suitable for the determination of the seven bisphenols in sediment.

双酚A(BPA)于1891年首次被合成,于20世纪50年代开始商业化生产^[1]^。BPA是世界上最常用和产量最高的化学品之一,广泛应用于聚碳酸酯塑料和环氧树脂行业^[2]^。由于BPA的广泛使用及其潜在毒性^[3,4]^,加拿大(2008年)、美国(2010年)、中国(2011年)和欧盟(2011年)等国家和地区已限制或禁止在婴儿奶瓶中使用BPA,法国于2015年1月起禁止在任何食品或饮料包装中使用BPA^[5]^。双酚B(BPB)、双酚S(BPS)、双酚F(BPF)和双酚AF(BPAF)等双酚类似物(bisphenol analogues),已经作为BPA的替代品而大量使用^[6]^。但是,双酚类似物可能具有与BPA相似或更大的毒性作用^[7,8]^。双酚类化合物(bisphenols)由于广泛使用而普遍存在于水体、土壤、沉积物等环境介质中,但其含量较低^[9,10]^。

双酚类化合物具有不同的物理化学性质,且在环境介质中的含量较低,导致环境样品中双酚类化合物的分析很困难^[11]^。样品的有效预处理(一般包括提取和纯化)对于复杂介质中低含量目标化合物的检测至关重要^[12]^。固体样品中目标化合物的提取包括从固体颗粒上解吸、在颗粒孔隙内扩散以及进入溶剂主体3个过程^[13]^。沉积物中双酚类化合物常用的预处理方法包括超声离心^[14⇓-16]^、振荡超声^[17]^、振荡离心^[18]^、加速溶剂萃取(ASE)^[19]^和固相萃取^[20]^等,常用的检测仪器有气相色谱-串联质谱(GC-MS/MS)^[21⇓-23]^、液相色谱(LC)^[24,25]^和液相色谱-串联质谱(LC-MS/MS)^[26]^。双酚类化合物具有较高的极性,GC-MS通常需要复杂的衍生化^[27]^。LC-MS因其多功能性、特异性、选择性和无需衍生化等优点,可以检测环境中较低含量的双酚类化合物^[28]^。Yang等^[15]^建立了超声离心-固相萃取净化-LC-MS/MS法用于测定沉积物中的BPS、BPF、BPA、BPB、BPAF、四氯双酚A和四溴双酚A。Wang等^[17]^建立了振荡超声-固相萃取净化-GC-MS法用于测定沉积物中的BPS、BPF、BPA、BPB、BPAF、双酚Z(BPZ)和双酚AP(BPAP)。贺小敏等^[18]^建立了振荡离心-固相萃取净化-LC-MS/MS法用于测定沉积物中的BPA、BPS、雌酮、*β*-雌二醇、雌三醇和17*α*-乙炔雌二醇等11种环境激素。Xu等^[19]^建立了ASE-旋转蒸发-LC-MS/MS法用于测定土壤中的BPS、BPF、BPA、BPB和BPAF等13种双酚类似物。超声处理和振荡处理常用于从固体样品中提取分析物,但由于其需要大量的有害有机溶剂和较长的提取时间,已逐步被微波辅助萃取、超临界流体萃取和加速溶剂萃取等所取代^[29]^。ASE是一种在高温高压下使用有机溶剂从固体或半固体样品中提取物质的自动化方法^[30]^。高温高压有助于溶剂渗透到样品基质中,最大限度地与分析物接触,提高了提取效率^[31,32]^。与传统萃取方法相比,ASE具有自动化、试剂用量少、效率高、回收率高和重现性好等优点^[33]^,已成为从固体环境样品、生物基质和食品中提取有机化合物的常用方法^[34]^。但是,加速溶剂萃取后需要采用固相萃取进行萃取液净化程序,以防其他物质干扰分析^[35]^。

目前,尚未建立沉积物中双酚类化合物的标准检测方法。因此,有必要建立高效、灵敏、准确的沉积物中双酚类化合物的检测方法,为沉积物中双酚类化合物赋存状况及潜在风险研究提供技术支撑。本文在已有研究基础上,建立了加速溶剂萃取-固相萃取净化结合超高效液相色谱-串联质谱法测定沉积物中7种双酚类化合物(BPA、BPB、BPF、BPS、BPZ、BPAF和BPAP)的检测方法。通过对色谱流动相和质谱参数进行优化,采用正交试验确定了加速溶剂萃取的萃取溶剂、萃取温度和循环次数等条件,评估了该方法的回收率、精密度、准确度、基质效应(ME)和检出限,并分析了骆马湖湖区及其入湖河流沉积物中7种双酚类化合物的赋存特征。

## 1 实验部分

### 1.1 仪器、材料与试剂

超高效液相色谱仪(Waters Acquity,美国Waters公司);三重四极杆复合线性离子阱质谱仪(QTrap 5500,美国Sciex公司);加速溶剂萃取仪(Dionex ASE 350,美国Thermo Fisher公司);自动固相萃取仪(Aqua Trace ASP E900,日本岛津公司);台式冷冻干燥机(Bench TopPro,美国SP Scientific公司);臼式研磨仪(MG 200,北京格瑞德曼仪器设备有限公司);电子天平(BSA 124S,德国Sartorius公司); Milli-Q超纯水系统(IQ 7000,德国Merck公司); Oasis HLB固相萃取柱(600 mL/200 mg)和针头过滤器(0.22 μm,美国Waters公司)。

甲醇、乙腈和乙酸乙酯(均为色谱纯)购于德国Merck公司。硅藻土购于美国Thermo Fisher公司。氨水和乙酸铵(均为分析纯)购于上海国药集团。BPA(纯度99.6%)、BPAF(纯度100%)、BPAP(纯度99.5%)、BPB(纯度98.4%)、BPZ(纯度100%)、BPS(纯度98.0%)和BPF(纯度100%)标准储备溶液购于美国AccuStandard公司,内标BPA-d_4_(纯度99.0%)购于美国First Standard公司。7种双酚类化合物的详细信息见表1。

**表1:** 7种双酚类化合物的化学式、CAS号、lg *K*_ow_和结构式

Bisphenol	Formula	CAS No.	lg *K*_ow_^*^	Structural formula
BPA	C_15_H_16_O_2_	80-05-7	3.64	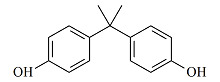
BPB	C_16_H_18_O_2_	77-40-7	4.13	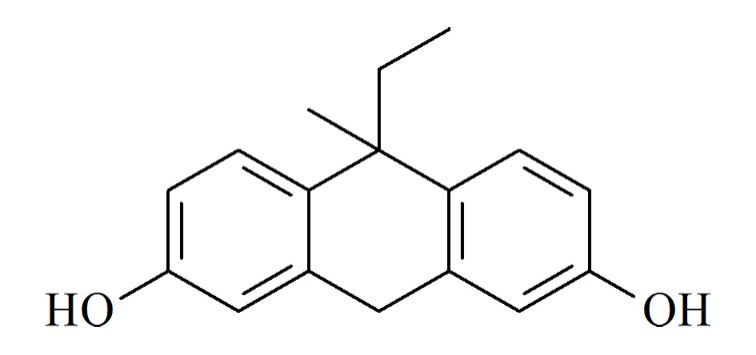
BPF	C_13_H_12_O_2_	620-92-8	3.06	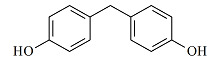
BPS	C_12_H_10_O_4_S	80-09-1	1.65	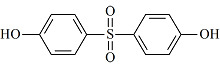
BPZ	C_18_H_20_O_2_	843-55-0	5.48	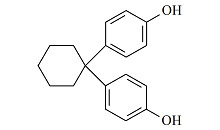
BPAF	C_15_H_10_F_6_O_2_	1478-61-1	4.47	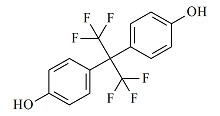
BPAP	C_20_H_18_O_2_	1571-75-1	4.86	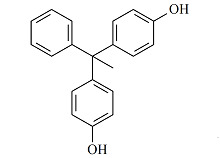

* From USEPA EPI Suite software (https://www.epa.gov/).

### 1.2 样品采集和保存

根据国家环境保护标准HJ 494-2009《水质采样技术指导》,于2020年7月采用不锈钢抓斗式采泥器采集了14个骆马湖湖区沉积物样品(深度0~20 cm)和17个入湖河流沉积物样品(深度0~20 cm)。骆马湖湖区样品在船上采集,入湖河流样品在河岸采集。采集的样品置于棕色玻璃瓶密封保存,立即转移至实验室,置于-20 ℃冷冻保存。

### 1.3 样品前处理

沉积物样品的前处理采用ASE方法。称取5.000 g经冷冻干燥、研磨、过100目尼龙筛的沉积物样品,将其与5.000 g硅藻土混匀后加入底部用纤维素滤膜覆盖的萃取池中。萃取条件如下:萃取剂为乙腈,压力为10.3 MPa (1500 psi),温度为100 ℃,预加热时间2 min,加热时间5 min,静态萃取时间5 min, 60%的萃取池体积作为冲洗容量,氮气吹扫120 s,静态循环3次,将萃取液收集至收集瓶中。

沉积物样品净化方法采用本课题组之前建立的固相萃取方法^[36]^,使用全自动固相萃取仪以5.0 mL/min的流速依次用10 mL甲醇和10 mL超纯水活化萃取柱,向沉积物萃取液中加入超纯水至500 mL,混匀,用3 mol/L的H_2_SO_4_调节pH至3,以8.0 mL/min的流速通过活化后的萃取柱,用10 mL 10%甲醇水溶液淋洗萃取柱,将淋洗后的萃取柱在真空泵负压下抽干60 min后,使用9.0 mL甲醇洗脱萃取柱。在40 ℃水浴条件下,用N_2_将洗脱下来的样品吹至体积小于1 mL,最后用甲醇复溶至1 mL。用0.22 μm针头过滤器过滤,待上机分析。

### 1.4 色谱-质谱条件

#### 1.4.1 色谱条件

Acquity UPLC BEH C_18_色谱柱(100 mm×2.1 mm, 1.7 μm);以0.05%(v/v)氨水(A)和乙腈(B)作为流动相,进样体积为2 μL;流速:0.3 mL/min;柱温:40 ℃。梯度洗脱程序:0~2 min, 60%A; 2~6 min, 60%A~40%A; 6~6.5 min, 40%A; 6.5~7 min, 40%A~60%A; 7~8 min, 60%A。

#### 1.4.2 质谱条件

电喷雾离子源;负离子模式(ESI^-^);离子源喷雾电压:-4500 V;雾化器(GS 1)压力:60.0 kPa;辅助气(GS 2)压力:65.0 kPa;气帘气(CUR)压力:35.0 kPa;离子源温度(TEM): 550 ℃;碰撞气电压(CAD): Medium。其他参数见表2。

**表2:** 目标化合物分析的质谱参数

Bisphenol	Retention time/min	Parent ion (*m/z*)	Daughter ion (*m/z*)	DP/ V	CE/ eV
BPA	2.32	227.1	211.1^*^	-136	-27
			133.0	-108	-32
BPA-d_4_	2.33	231.0	135.1	-132	-34
			215.0	-134	-29
BPB	2.96	241.1	211.1^*^	-123	-30
			225.9	-106	-23
BPF	1.87	199.1	105.0^*^	-121	-27
			92.8	-121	-30
BPS	1.31	248.4	107.9^*^	-129	-34
			92.1	-121	-38
BPZ	4.15	267.1	173.0^*^	-142	-34
			145.0	-121	-48
BPAF	2.17	335.0	264.5^*^	-130	-30
			197.0	-139	-50
BPAP	3.65	289.0	273.9^*^	-117	-21
			195.1	-117	-35

* Quantitative ion. DP: declustering potential; CE: collision energy.

## 2 结果与讨论

### 2.1 分析条件的优化

#### 2.1.1 色谱条件的优化

7种双酚类化合物均含有酚羟基,具有相似的结构,采用较高硅羟基活性的色谱柱,可能导致目标化合物色谱峰扁平和拖尾^[18]^。C_18_色谱柱采用较低硅羟基活性填料,且在广泛的pH值范围内具有良好的化学稳定性,因此本研究采用Acquity UPLC BEH C_18_色谱柱(100 mm×2.1 mm, 1.7 μm)作为色谱分离柱。

流动相应具有黏度低、与检测器相容性好、低毒等特点。在流动相中加入适当的有机酸或缓冲盐可以提高目标化合物的电离效率,提高分析方法的灵敏度^[37]^。本研究考察了水-乙腈、5 mmol/L醋酸铵-乙腈和0.05%(v/v)氨水-乙腈3组流动相对7种双酚类化合物的分离效果、色谱峰形状和响应值的影响。实验结果显示,在3组流动相中,0.05%(v/v)氨水-乙腈为流动相时,进样量为2 μL, 7种双酚类化合物的响应较高、峰形尖、无拖尾和基线无漂移。这是因为7种双酚类化合物含有弱酸性酚羟基,在流动相中加入呈碱性的氨水,有助于双酚类化合物产生[M-H]^-^前体离子^[38]^。因此后续选用0.05%(v/v)氨水(A)-乙腈(B)作为流动相,进样体积为2 μL,流速为0.3 mL/min,柱温为40 ℃。梯度洗脱程序基于Yan等^[14]^的方法进行优化,优化后的梯度程序见1.4.1节。在此条件下,7种双酚类化合物的总离子流色谱图见图1。

**图1:**
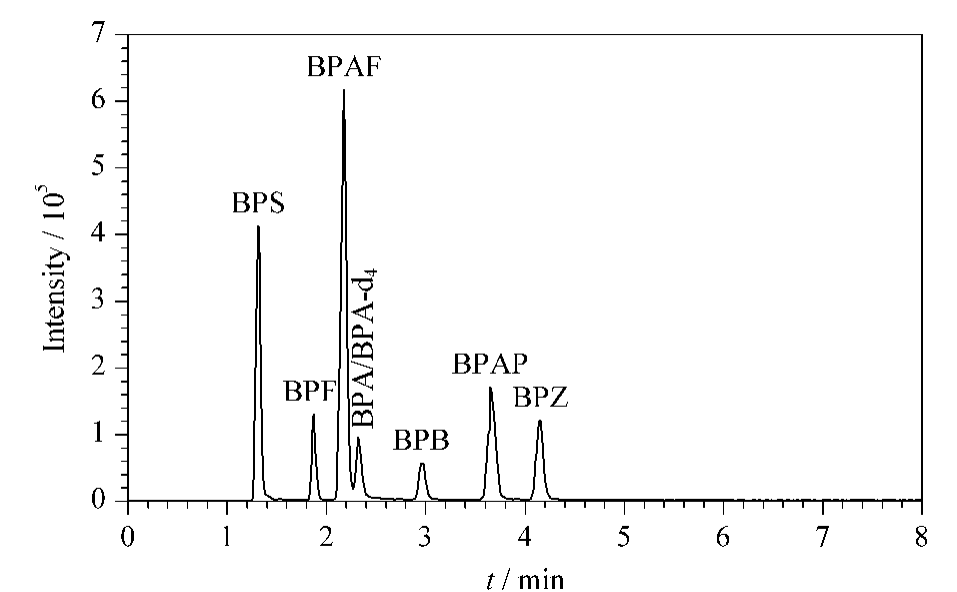
7种双酚类化合物(50 μg/L)的总离子流色谱图

#### 2.1.2 质谱分析条件

7种双酚类化合物和内标物均含有酚羟基,容易损失质子,因此电喷雾离子源采用ESI^-^模式。采用针泵恒流进样方式,分别将100 μg/L的单一标准品以10 μL/min的流速注入离子源。在全扫描模式下选择适当的分子离子峰,采用子离子扫描确定化合物的定量离子和定性离子,并优化碰撞电压和去簇电压。7种双酚类化合物的质谱参数见表2。

### 2.2 沉积物样品前处理的优化

萃取溶剂是ASE提取过程中的重要因素^[39]^,应依据目标化合物的极性和样品的基质成分来选择。萃取温度是ASE最重要的参数,升高萃取温度可增加分析物的溶解度和扩散速率,减弱或破坏分析物和基质成分之间的相互作用力,降低溶剂的黏度和表面张力^[40]^。静态萃取循环次数对萃取效率具有显著的影响。增加静态萃取循环次数可以不断引入新鲜溶剂,使萃取更加彻底^[41]^。

为了获取ASE提取沉积物中7种双酚类化合物的最优萃取条件,以空白沉积物为实验对象,在7种双酚类化合物的添加量分别为20 ng/g的条件下,进行加标回收试验。将萃取溶剂(A)、萃取温度(B)和循环次数(C)作为3个因素,每个因素3个水平,根据正交设计表L_9_(3^3^)设计三因素三水平的正交试验。以7种双酚类化合物回收率为指标,研究萃取溶剂(甲醇、乙腈、乙酸乙酯)、萃取温度(80、100和120 ℃)和循环次数(1次、2次和3次)对7种双酚类化合物回收率的影响,每组实验重复3次。正交试验设计和结果如表3、表4所示。

**表3:** 正交试验参数和回收率

No.	Factors					Recoveries/%							
	Extraction solvent		Extraction temperature/ ℃	Cycle number		BPA	BPB		BPF	BPS	BPZ	BPAF	BPAP
1		methanol	80	1		56.1	52.6		53.8	37.7	53.3	49.6	42.8
2		methanol	100	2		64.9	61.8		64.6	47.4	55.8	51.1	48.6
3		methanol	120	3		66.4	56.3		60.4	51.1	60.7	58.1	40.5
4		acetonitrile	80	3		97.9	90.6		101.6	64.3	76.2	88.4	75.2
5		acetonitrile	100	1		103.3	94.2		89.2	68.7	84.8	79.2	82.7
6		acetonitrile	120	2		91.4	87.9		94.5	71.7	78.7	82.1	72.2
7		ethyl acetate	80	2		41.4	43.2		45.4	26.5	37.7	31.4	38.5
8		ethyl acetate	100	3		54.4	40.6		51.5	38.3	45.4	37.5	31.5
9		ethyl acetate	120	1		44.9	37.4		39.2	31.4	41.3	44.6	26.2

**表4:** 正交试验结果

Bisphenol	Item	Factors		
		Extraction solvent	Extraction temperature/℃	Cycle number
BPA	${\bar{K}}_{1}$	62.5	65.1	68.1
	${\bar{K}}_{2}$	97.5	74.2	65.9
	${\bar{K}}_{3}$	46.9	67.6	72.9
	*R*	50.6	9.1	7.0
BPB	${\bar{K}}_{1}$	56.9	62.1	61.4
	${\bar{K}}_{2}$	90.9	65.5	64.3
	${\bar{K}}_{3}$	40.4	60.5	62.5
	*R*	50.5	5.0	2.9
BPF	${\bar{K}}_{1}$	59.6	66.9	60.7
	${\bar{K}}_{2}$	95.1	68.4	68.2
	${\bar{K}}_{3}$	45.4	64.7	71.2
	*R*	49.7	3.7	10.4
BPS	${\bar{K}}_{1}$	45.4	42.8	45.9
	${\bar{K}}_{2}$	68.2	51.5	48.5
	${\bar{K}}_{3}$	32.1	51.4	51.2
	*R*	36.2	8.6	5.3
BPZ	${\bar{K}}_{1}$	56.6	55.7	59.8
	${\bar{K}}_{2}$	79.9	62.0	57.4
	${\bar{K}}_{3}$	41.5	60.2	60.8
	*R*	38.4	6.3	3.4
BPAF	${\bar{K}}_{1}$	52.9	56.5	57.8
	${\bar{K}}_{2}$	83.2	55.9	54.9
	${\bar{K}}_{3}$	37.8	61.6	61.3
	*R*	45.4	5.7	6.5
BPAP	${\bar{K}}_{1}$	44.0	52.2	50.6
	${\bar{K}}_{2}$	76.7	54.3	53.1
	${\bar{K}}_{3}$	32.1	46.3	49.1
	*R*	44.6	8.0	4.0

$\bar{K_{i}}$ : average recovery for the same level *i*, *i*=1, 2, 3; *R*: range, *R*= ${\bar{K}}_{imax}$ - ${\bar{K}}_{imin}$ .

表4显示了3个因素对7种双酚类化合物回收率的影响,*R*值越大代表该因素对回收率的影响越大。在3个因素中,萃取溶剂对7种双酚类化合物的回收率影响最大,萃取温度对BPA、BPB、BPS、BPZ和BPAP回收率影响次之,循环次数对回收率影响最小,即因素A>因素B>因素C。相比上述5种双酚类化合物,循环次数对BPF和BPAF回收率的影响大于萃取温度,即因素A>因素C>因素B。 $\bar{K_{i}}$ 值越大代表在该水平下目标化合物回收率越好,BPA、BPF、BPS和BPZ最优组合为A_2_B_2_C_3_, BPB和BPAP最优组合为A_2_B_2_C_2_, BPAF的最优组合为A_2_B_3_C_3_。然而在优化的3个因素中,循环次数对BPB和BPAP的回收率影响最小,萃取温度对BPAF回收率影响最小。综合考虑极差分析结果,最终确定最优组合为A_2_B_2_C_3_,即萃取溶剂为乙腈,萃取温度为100 ℃,循环次数为3次。

### 2.3 方法性能

#### 2.3.1 基质效应

基质效应由Kebarle和Tang首先提出^[42]^。基质是指样品中除目标分析物以外的成分^[43]^。在采用LC-MS/MS分析环境样品时,分析物的信号强度可能在基质中受到抑制或增强^[44]^。基质效应会对方法的重现性和准确性产生影响^[45,46]^。本文中基质效应根据Matuszewski等^[47]^提出的方法进行计算。


(1) ME=*B/A*×100%

式中,*A*是以甲醇为溶剂的标准溶液的响应值,*B*是以不含待测物的萃取液为溶剂,加入相同含量的标准溶液后的响应值。ME为100%时,没有基质效应;ME大于100%时,电离化增强;ME小于100%时,电离化受到抑制^[48]^。ME在-20%~20%内认为没有基质影响^[49]^。

用甲醇制备7种双酚类化合物的混合标准溶液;采用优化后的方法制备空白基质样品,用空白基质溶液配制与混合标准溶液相同浓度的标准溶液;采用UPLC-MS/MS测定上述两种溶液。如表5所示,BPA、BPF、BPAF的基质效应分别为112.4%、102.5%、106.3%,表明BPA、BPF和BPAF存在基质增强效应。BPB、BPS、BPZ、BPAP的基质效应分别为89.3%、82.7%、93.4%、87.2%,表明BPB、BPS、BPZ、BPAP存在基质抑制效应。

**表5:** 沉积物中7种双酚类化合物的线性关系、LOD、LOQ、ME和回收率(*n*=3)

Bisphenol	Regression equation	*r*^2^	LOD/(ng/g)	LOQ/(ng/g)	Spiked/(ng/g)	Recovery/%	RSD/%	ME/%
BPA	*y*=3344.8*x*-3055.1	0.9992	0.3	1.2	2.0	92.6	8.4	112.4
					10	102.8	9.2	
					20	97.7	7.8	
BPB	*y*=43092*x*-38319	0.9995	0.1	0.4	2.0	80.7	7.7	89.3
					10	85.1	8.3	
					20	90.4	8.5	
BPF	*y*=52079*x*-54265	0.9993	0.2	0.8	2.0	82.8	6.2	102.5
					10	84.3	9.4	
					20	93.7	10.3	
BPS	*y*=359131*x*-363426	0.9994	0.02	0.08	2.0	74.9	9.2	82.7
					10	78.4	8.5	
					20	77.9	7.6	
BPZ	*y*=59535*x*-45969	0.9991	0.04	0.16	2.0	78.2	8.7	93.4
					10	82.3	9.7	
					20	86.8	7.4	
BPAF	*y*=488005*x*-454159	0.9993	0.01	0.04	2.0	78.9	9.7	106.3
					10	80.2	8.6	
					20	83.7	9.4	
BPAP	*y*=307737*x*-304950	0.9992	0.05	0.2	2.0	75.4	8.2	87.2
					10	79.1	9.1	
					20	81.9	8.7	

Linear range: 1.0-200 μg/L; *y*: the ratio of the peak areas of an analyte to internal standard; *x*: mass concentration of the analyte, μg/L.

#### 2.3.2 方法性能

依照欧盟SANTE/12682/2019中的规定评估了线性范围、精密度和准确度。所有实验重复3次。分析空白样品以检查是否存在仪器和样品污染^[11]^。每10个样品执行1个试剂空白以监测仪器背景^[50,51]^。每20个样品插入1个混合标准溶液(50 μg/L)作为质控样品。采用内标法进行定量分析。分别配制1.0、2.0、5.0、10、20、50、100和200 μg/L的7种双酚类化合物的混合标准溶液,各梯度的标样中均含50 μg/L内标,以分析物色谱峰面积与内标色谱峰面积的比值(*y*)和分析物的质量浓度(*x*)作线性回归分析,得出线性方程和相关系数(*r*^2^)。7种双酚类化合物在1.0~200 μg/L内线性关系良好,线性相关系数均大于0.999。

按照1.3节方法进行7种双酚类化合物的空白加标回收试验,在空白沉积物样品中添加低、中、高3个水平的7种双酚类化合物,使得各加标样品中7种双酚类化合物的含量分别为2.0、10和20 ng/g,采用优化后的方法对样品进行处理,每个实验重复3次,计算加标回收率和相对标准偏差(RSD)。添加量为2.0 ng/g时,回收率为74.9%~92.6%, RSD为6.2%~9.7%;添加量为10 ng/g时,回收率为78.4%~102.8%, RSD为8.3%~9.7%;添加量为20 ng/g时,回收率为77.9%~97.7%, RSD为7.4%~10.3%。使用3倍和10倍信噪比(*S/N*)分别确定7种双酚类化合物的检出限(LOD)和定量限(LOQ)^[52]^。当沉积物样品为5.0 g时,浓缩后的体积为1 mL,进样体积为2 μL时,该方法7种双酚类化合物的检出限为0.01~0.3 ng/g,定量限为0.04~1.2 ng/g。沉积物中7种双酚类化合物的LOD、LOQ、ME和回收率见表5。

将本研究建立的检测方法与现有文献进行了比较,见表6。本研究中BPF、BPS、BPZ、BPAF、BPAP的检出限低于Xu等^[19]^的研究;BPS、BPAF的检出限低于Yang等^[15]^的研究,BPF的检出限与Yang等^[15]^的研究相同;BPS、BPZ、BPAF、BPAP的检出限低于Wang等^[17]^的研究。本研究中BPA和BPS的检出限高于贺小敏等^[18]^的研究。

**表6:** 与类似分析方法的比较

Sample	Bisphenols	Analytical methods	Linear range/ (μg/L)	LOD/ (ng/g)	Recovery/ %	Ref.
Sediment	BPS, BPF, BPA, BPB, BPZ, BPAF, BPAP	ASE-SPE-LC-MS/MS	1.0-200	0.01-0.3	74.9-102.8	this study
Sediment	BPS, BPF, BPA, BPB, BPZ, BPAF, BPAP	ultrasound-assisted-SPE-GC-MS	-	0.03-0.32	89.3-105	[17]
Soil	BPS, BPF, BPA, BPB, BPZ, BPAF, BPAP	ASE-rotary evaporator-LC-MS/MS	1.0-200	0.03-0.39	80-110	[19]
Sediment	BPS, BPF, BPA, BPB, BPAF	ultrasonic-SPE-LC-MS/MS	-	0.02-0.2	66.1-103.2	[15]
Sediment	BPA	oscillatory-SPE-LC-MS/MS	1.0-200	0.05	103.5-119.7	[18]
	BPS		1.0-100	0.01	86.1-98.0	

-: not mentioned.

### 2.4 实际样品的应用

采用建立的检测方法分析了14个骆马湖湖区沉积物样品和17个入湖河流沉积物样品中的7种双酚类化合物,检出率和含量范围见表7。实际样品中双酚类化合物的色谱图见图2。骆马湖湖区沉积物中检测出BPA、BPB、BPF、BPS和BPAF,入湖河流沉积物中检测出BPA、BPF和BPS。在骆马湖湖区和入湖河流沉积物中,BPA和BPF的检出率均为100%; BPA的含量范围分别为11.9~27.7 ng/g和14.0~38.0 ng/g, BPF的含量范围分别为11.0~19.5 ng/g和11.3~27.3 ng/g。在骆马湖湖区和入湖河流沉积物中BPS的检出率分别为42.9%和17.6%,含量范围均为ND~0.2 ng/g。

**表7:** 骆马湖及入湖河流沉积物中7种双酚类化合物的检出率和含量范围

Bisphenol	Luoma Lake (*n*=14)			Inflow rivers (*n*=17)	
	Detection frequency/ %	Content range/ (ng/g)		Detection frequency/ %	Content range/ (ng/g)
BPA	100	11.9-27.7		100	14.0-38.0
BPB	14.3	ND-0.6		0	ND
BPF	100	11.0-19.5		100	11.3-27.3
BPS	42.9	ND-0.2		17.6	ND-0.2
BPZ	0	ND		0	ND
BPAF	7.1	ND-0.1		0	ND
BPAP	0	ND		0	ND

ND: not detected; *n*: number of sampling points.

**图2:**
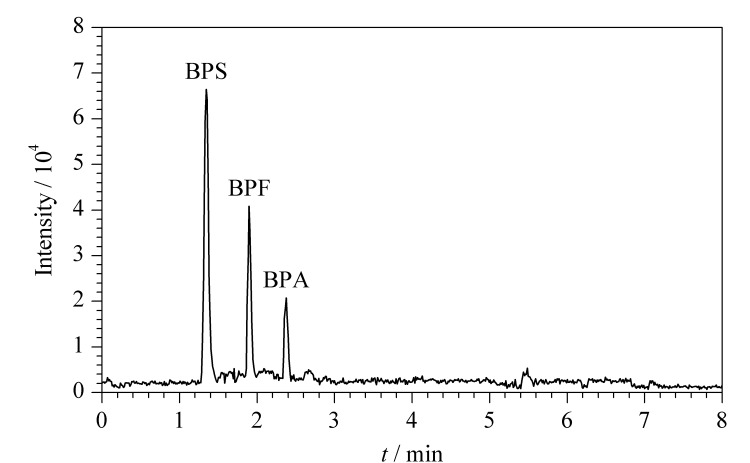
实际样品中双酚类化合物的色谱图

与文献检测结果相比,骆马湖沉积物中的BPA含量(11.9~27.7 ng/g)低于中国的珠江(1.5×10^2^~7.2×10^2^ ng/g)^[16]^、东江(29.8~2.0×10^3^ ng/g)^[16]^和太湖(3.63~2.7×10^2^ ng/g)^[53]^,美国的Rivers and bays(ND~1.1×10^2^ ng/g)^[54]^和韩国的Lake Shihwa(ND~1.4×10^4^ ng/g)^[54]^,但高于中国的浑河(0.15~2.1 ng/g)^[55]^、辽河(ND~0.45 ng/g)^[55]^和日本的Tokyo bay(1.9~23.0 ng/g)^[54]^。骆马湖沉积物中的BPF含量(11.0~19.5 ng/g)低于中国的珠江(26.7~3.4×10^2^ ng/g)^[16]^、东江(51.4~1.4×10^3^ ng/g)^[16]^、太湖(3.02~95.2 ng/g)^[53]^和韩国的Lake Shihwa(ND~9.6×10^3^ ng/g)^[54]^,但高于中国的浑河(ND~3.8 ng/g)^[55]^、辽河(ND~0.41 ng/g)^[55]^和日本的Tokyo bay(ND~9.1 ng/g)^[54]^。骆马湖沉积物中的BPS含量(ND~0.2 ng/g)低于韩国的Lake Shihwa(ND~2.0×10^3^ ng/g)^[54]^。

## 3 结论

本文建立了加速溶剂萃取-固相萃取净化结合超高效液相色谱-串联质谱法测定沉积物中7种双酚类化合物的检测方法。通过正交试验优化确定了ASE提取沉积物中7种双酚类化合物的参数。该方法简便、快速,准确度和精密度较高,适用于沉积物中7种双酚类化合物的检测。
